# A descriptive study of the trends of COVID-19 in Zimbabwe from March-June 2020: policy and strategy implications

**DOI:** 10.11604/pamj.supp.2020.37.1.25835

**Published:** 2020-11-09

**Authors:** Grant Murewanhema, Trouble Burukai, Dennis Mazingi, Fabian Maunganidze, Jacob Mufunda, Davison Munodawafa, William Pote

**Affiliations:** 1 Department of Obstetrics and Gynaecology, College of Health Sciences, University of Zimbabwe, Harare, Zimbabwe,; 2 Department of Anatomy, Faculty of Medicine, Midlands State University, Gweru, Zimbabwe,; 3 Department of Surgery, College of Health Sciences, University of Zimbabwe, Harare, Zimbabwe,; 4 Department of Physiology, Faculty of Medicine, Midlands State University, Gweru, Zimbabwe,; 5 Ethnobiology-Based Drug Discovery, Research and Development Trust, Gweru, Zimbabwe,; 6 Department of Community Medicine, Faculty of Medicine, Midlands State University, Gweru, Zimbabwe

**Keywords:** Coronavirus disease 2019 (COVID-19), pandemic, Zimbabwe, severe acute respiratory syndrome coronavirus 2, infection

## Abstract

**Introduction:**

the first cases of COVID-19 were reported in China in December 2019. Since then, the disease has evolved to become a global pandemic. Zimbabwe reported its first case on 20^th^ March 2020, and the number has been increasing steadily. However, Zimbabwe has not witnessed the exponential growth witnessed in other countries so far, and the trajectory seems different. We set out to describe the epidemiological trends of COVID-19 in Zimbabwe from when the first case was confirmed to June 2020.

**Methods:**

data were collected from daily situation reports that were published by the Zimbabwean Ministry of Health and Child Care from 20^th^ March to 27^th^ June 2020. Missing data on the daily situation reports was not imputed.

**Results:**

as of 27^th^ June 2020, Zimbabwe had 567 confirmed COVID-19 cases. Eighty-two percent of these were returning residents and 18% were local transmission. The testing was heavily skewed towards returnees despite a comprehensive testing strategy. Of the confirmed cases, 142 were reported as recovered. However, demographic data for the cases were missing from the reports. It was not possible to estimate the probable period of infection of an active case, and case fatality in Zimbabwe was about 1% for the first 4 months of the pandemic.

**Conclusion:**

the epidemiological trends of COVID-19 experienced in Zimbabwe between March and June 2020 are somewhat different from what has been observed elsewhere. Further research to determine the reasons for the differences is warranted, to inform public health practice and tailor make suitable interventions.

## Introduction

COVID-19 is a contagious zoonotic disease caused by a novel coronavirus, named the Severe Acute Respiratory Syndrome Coronavirus-2 (SARS-CoV-2) [1,2]. From a sudden outbreak in Wuhan, China, in December 2019 to a worldwide pandemic, massively affecting countries like Italy, Spain and USA in less than 3 months [3], the disease has spread to all parts of the world, including Zimbabwe. The spread to various parts of the world has been in an asynchronous pattern, most likely due to multiple contagion sources [3]. The World Health Organisation (WHO) declared COVID-19 a global health emergency on 30 January 2020, before characterising it as a pandemic on 11^th^ March 2020 [4].

Within weeks of onset, COVID-19 had spread to over 100 countries across the world. By the end of June 2020, over 10 million cases had been reported to the WHO, with over 500 000 fatalities [5]. The WHO regional situation reports indicate that by 1 July 2020, the American region had reported an estimated 5,218,590 confirmed cases, making it the most affected continent [5]. Europe, Eastern Mediterranean, South-East Asia, Africa and the Western Pacific followed in descending order. The African continent is ranked the fifth most affected region according to the WHO as of 1 July 2020. South Africa was leading as of 1 July 2020, with 151 209 cases and 2 657 deaths, followed by Egypt with 68 311 cases and 2 957 deaths [5,6]. The spread of the virus may continue until an effective vaccine is found.

Zimbabwe confirmed its first case of COVID-19 on 20^th^ March 2020 [7]. The case was a 38-year-old male resident of Victoria Falls. He had travelled to the United Kingdom on 7^th^ March 2020 and returned to the country on 15^th^ March 2020. It was also reported that a British tourist who had visited Victoria Falls earlier that week had tested positive for COVID-19 upon returning to the United Kingdom. Zimbabwe declared a 21-day national lockdown starting on 30^th^ March 2020. By 25^th^ May 2020, two months after the first case had been confirmed; Zimbabwe had 56 confirmed cases, including 27 active cases, 4 deaths and 25 recoveries. Whilst the Ministry of Health and Child Care (MOHCC) produces daily situation reports, and regular surveillance updates are done, no review of trends from the onset of the outbreak in Zimbabwe has been published. This review aims to describe the epidemiological trends of the disease in Zimbabwe from the onset up to 27^th^ June 2020.

## Methods

Data were extracted from publicly available daily situation reports published by the MOHCC from 20^th^ March to 27^th^ June 2020. The data extracted included the number of tests done, confirmed cases, mortalities, recovered cases, active cases, local cases, imported cases, and the countries where the returnees were coming from. Data on the distribution of the COVID-19 cases within the provinces of Zimbabwe were also collected. Missing data on the daily situation reports was not looked for. Data from these reports were transcribed to a database in Microsoft Excel® and exported to Stata® Version 14.1 for statistical analysis.

**Statistical analysis:** descriptive statistics were used for demographic data and graphs were generated to show characteristics and trends including country of origin of confirmed cases, demographic variables and time trends. All graphs were generated using Stata Version 14.1.

**Ethical considerations:** this is a description of data widely made available to the public, and therefore no ethical or institutional review board approval was necessary. The study did not involve individual participant data, and thus no informed consent was necessary.

## Results

About 50% of the tests done in Zimbabwe were rapid diagnostic tests (RDTs) (Table 1). Positive cases were confirmed by the reverse transcriptase polymerase chain reaction assays (RT-PCR), the diagnostic gold standard for SARS-CoV-2. Table 1 shows the summarized statistics for the COVID-19 outbreak in Zimbabwe from 20^th^ March 2020 to the 27^th^of June 2020.

**Table 1:** summary statistics for COVID-19 outbreak in Zimbabwe as of 27^th^ of June 2020

Variables	Total
Number of RT-PCR tests	29 641
Number of RDTs	36 597
Total number of combined tests	66 238
Number of deaths	6
Number of confirmed cases	567
Number of recovered cases	142
Number of active cases	419
Number of returnee COVID-19 cases	466
Number of local COVID-19 cases	101

**Figure 1:**
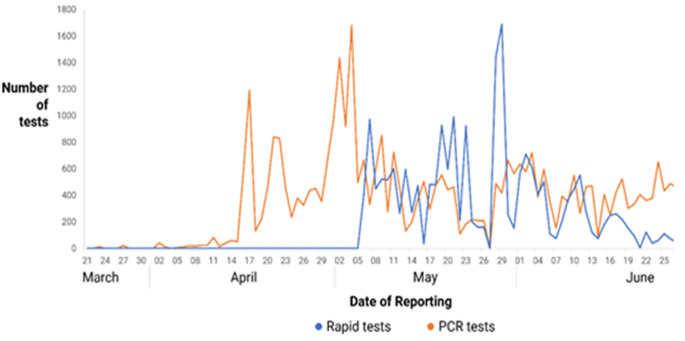
number of rapid diagnostic and RT-PCR tests done in Zimbabwe from March to June 2020

**Screening and diagnostic tests:** rapid diagnostic (RDTs) tests were used for screening, and RT-PCR for diagnosis of COVID-19. Initially, only RT-PCR assays were used. The RDTs were introduced towards the end of April, and their use increased initially but is now declining. A positive RDT was accompanied by a confirmatory RT-PCR. From 20^th^ March to 27^th^ June 2020, the number of RT-PCR assays done increased as shown in Figure 1. The number of RT-PCR assays done were fluctuant, with the highest number ever done on a single day being 1682. The total number of RT-PCR and RDT tests done during the period of this study in Zimbabwe was 66 238 (Table 1).

**Figure 2:**
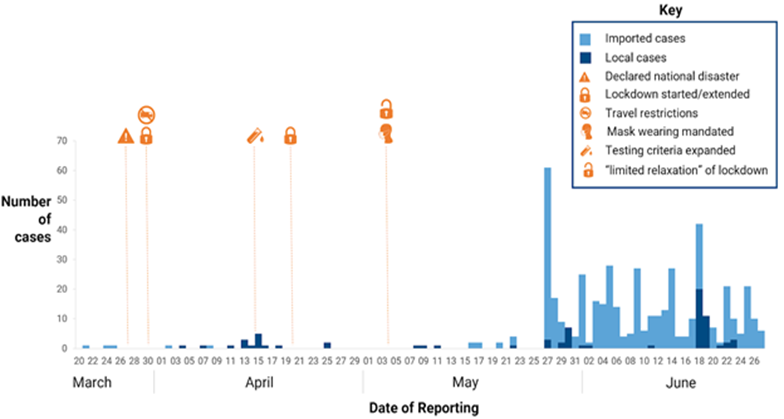
public health interventions introduced by the government as mitigating factors for COVID-19 in Zimbabwe between 20^th^ March and 27^th^ June 2020

**Mitigatory measures applied in Zimbabwe:**Figure 2 shows the public health interventions imposed by the government of Zimbabwe against the spread of COVID-19 in the country. Pertinent dates during the first three months of the pandemic are highlighted as well as the relevant public health measures instituted by the Government of Zimbabwe. During the initial lockdown period, the number of cases remained low. However, an increase in the number of cases was observed after relaxing of lockdown restrictions, despite the introduction of other measures such as the compulsory wearing of facemasks in public spheres.

**Figure 3:**
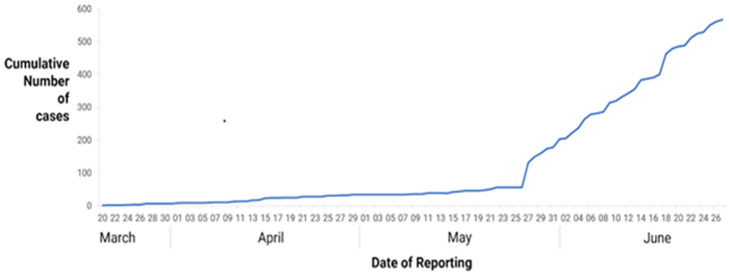
trends of COVID-19 between 20^th^ March and 27^th^ June 2020 in Zimbabwe

**COVID-19 positive cases:** numbers of confirmed COVID-19 cases remained low during the study period (Figure 3).

**Figure 4:**
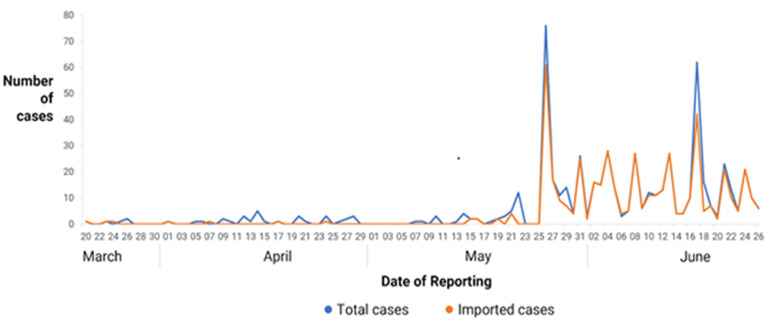
number of total positive and imported cases recorded daily from 20^th^ March to 26^th^ June 2020

**Returnee cases:** during the entire study period, 466 returnees tested positive for COVID-19, compared to 101 local transmissions (Table 1, Figure 4).

**Figure 5:**
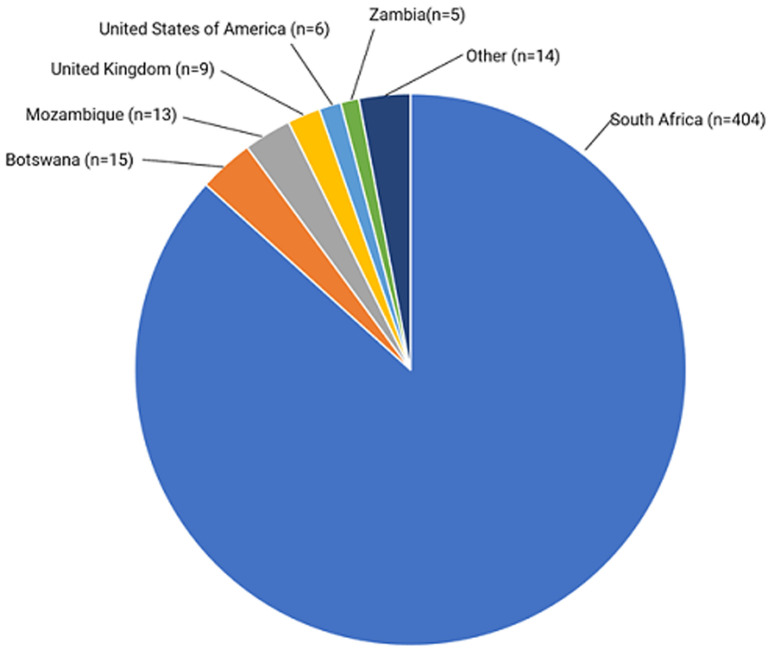
countries of origin of the COVID-19 positive Zimbabwean returnees; other countries are United Arab Emirates, South Sudan, France, South Korea, Tanzania, Malawi, Portugal, Lesotho and Namibia

**Source of imported cases:** the countries from where the confirmed COVID-19 positive returnees originated is shown in Figure 5. Eighty six point seven percent (86.7%) were from South Africa.

**Figure 6:**
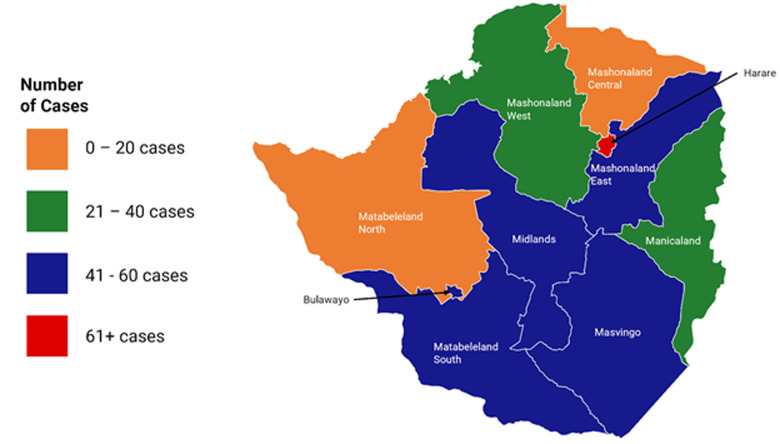
number of positive cases during the study period by province in Zimbabwe

**Spatial distribution of COVID-19 cases across Zimbabwe:**Figure 6 below shows the graphical mapping of confirmed COVID-19 cases in Zimbabwe during the period of the study. The majority of cases lie within the Harare Metropolitan Province, Bulawayo Metropolitan Province and the provinces that share borders with neighbouring South Africa and Botswana.

**Figure 7:**
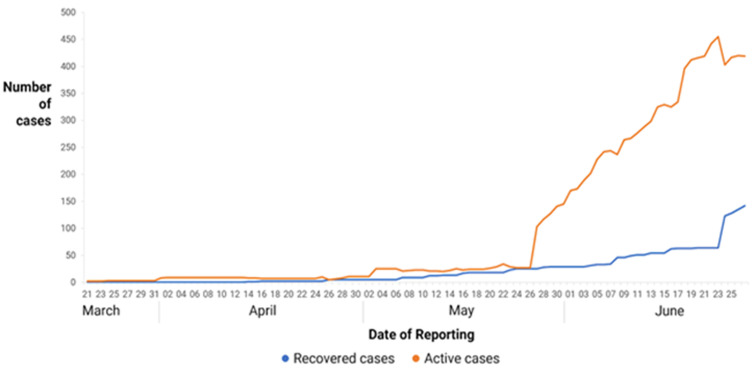
number of recovered and active cases from 20^th^ March to 27^th^ June 2020 in Zimbabwe

**Recovered and active cases:** the numbers of recovered and active cases are demonstrated in Figure 7.

## Discussion

Europe, America and Asia experienced exponential increases of disease burden once the COVID-19 outbreak commenced [1]. Their healthcare systems were rapidly overwhelmed, and the daily loss of lives exceeded 1000 over many instances. However, the pattern of disease in Africa took a different trajectory altogether. Scientists projected a grim outlook for Africa, given that countries in the developed world were struggling to cope. Scholarly projections by a team of researchers at the London School of Hygiene and Tropical Medicine, had predicted that Zimbabwe would reach 1000 infections by mid-April [8]. However, more than 3 months later, the country was still at about 60% of that projection. Zimbabwe confirmed the first case of COVID-19 on 20^th^ March 2020 [7] and since then; increases in caseload have not followed any predictable pattern. Isolated spikes in the detected cases occurred at some points. The first spike coincided with an expansion of the testing strategy, whilst the second spike coincided with a surge in the number of returnees from neighbouring countries who were mandatorily quarantined and tested [9]. During the initial lockdown, borders were entirely closed. At the time of analysis, the highest number of cases reported on any single day was 72, whereas South Africa and countries beyond were reporting over 1 000 cases per day [1,3].

Without evidence of widespread community transmission, Zimbabwe reserved testing for individuals meeting the case definition of symptomatic individuals with a history. With an increase in transmission of COVID-19 and influx of returning residents, an expanded testing strategy was introduced by the MOHCC on 14^th^ April 2020. This included testing of all hospitalised patients, patients with unexplained fever and community deaths. This removed the mandatory need for a history of travel, and expanding on the case definitions for COVID-19. Despite the government policy and strategy calling for increased testing, targets were not reached due to resource limitations. By the end of April 2020, out of the targeted 33 000 tests [9], only 8314 combined tests had been conducted. To mitigate against the testing limitations, serological RDTs were introduced. However, the role of RDTs in the management of COVID-19 remains undefined, for both antigen and antibody based assays. According to the WHO, RDTs should be useful for community surveillance and academic research, especially to assess the extent of community infection [10]. A recently published systematic review and meta-analysis by Bastos *et al*. supports this assertion, and casts doubts on the usefulness of the current serological assays [11]. In Zimbabwe, RDTs have been used mainly as screening assays and those who tested positive needed confirmatory RT-PCR testing [7,9]. Following the government of Zimbabwe´s declaration of COVID-19 as a national disaster on 27^th^ March 2020, a total lockdown was effected on 30^th^ March 2020. The burden of COVID-19 remained low; however, it is still difficult to attribute this just to lockdowns. It may also be argued that the testing strategy was not widely and correctly implemented. The majority of the testing has been concentrated on returnees in quarantine centres and points of entry, with very limited facility and community testing. It may therefore not be surprising that 92% of the cases have been detected among returnees. In fact, following the definitions of the patterns of transmission, Zimbabwe had not reached proper community transmission at the end of the study period, and at best, most of the local cases were either contact-traceable or sporadic. Community transmission implies the detection of multiple cases, or clusters of cases in communities, which are not directly epidemiologically linked to known imported cases [12].

The COVID-19 response included also the pillar addressing Risk Communication and Community Engagement (RCCE). The major focus for the RCCE activities is to promote physical and social distancing, personal hand hygiene (washing hands with soap or using an alcohol base sanitizer), protecting cough by coughing into the elbow, and the use of facemasks when one is in crowded places where one meter of physical space could be achieved. The RCCE activities also include information dissemination to increase awareness and positive behaviour change. This is achieved mostly through radio, television, posters and pamphlets and also interpersonal communication channels such as drama and community dialogues. As the RCCE pillar intensified its information dissemination on COVID-19, it would appear that communities are starting to show signs of information overload. There is low adherence to the WHO recommended preventive measures, namely social and physical distancing, washing hands frequently and use of masks. The contribution of human behaviour and complacency towards this may need to be evaluated adequately in social science studies. Other explanatory variables for increasing number of cases include increased numbers of returnees, porous borders and lack of adequate infection prevention and control measures in quarantine centres. Numerous personal, media and social media reports of inhumane and inappropriate living conditions in these centres have been circulated [13]. The majority of imported cases were from the neighbouring countries. South Africa contributed the most, with 404 cases as of 27^th^ June 2020. This has plausibility because South Africa is the most affected country on the African continent. Moreover, millions of Zimbabweans either live in South Africa or regularly travel to South Africa. These have been returning home to Zimbabwe because they lost their jobs, were locked in neighbouring countries when the lockdowns were initially imposed, or they have been deported for one reason or another. The increasing numbers of quarantine facility inmates testing positive has triggered debate on whether the individuals are coming into the country infected, or acquiring SARS-CoV-2 in these centres [13]. Nevertheless, upscaling of infection prevention and control measures is urgently needed in these centres to prevent those becoming hotspots for transmission in Zimbabwe.

The spatial distribution of the cases shows the greatest burden to be in Harare, Bulawayo and Matabeleland South Provinces, whilst Matabeleland North and Mashonaland Central Provinces had the lowest caseloads. Overall, the more urbanised provinces recorded the highest numbers of cases, whilst the less urbanised provinces reported the lowest. Overall, the more urbanised provinces recorded the highest numbers of cases, whilst the less urbanised provinces reported the lowest. On the other hand, Matabeleland South is in close proximity to South Africa, with concentration of imported cases in Beitbridge. In this regard, population density as well as proximity to a port of entry appear to be strongly linked to COVID-19 cases. Zimbabwe has a relatively young and rural population. It was hypothesized in a modelling study by Diop *et al*. that this could work favorably for Africa, with the youthful population being infected more but exhibiting asymptomatic or less severe disease, possibly modifying the severity of the epidemic [14]. However, this postulation from mathematical modelling is still to be substantiated by primary research, and calls for further analytical observational studies. The daily situation reports indicate that the majority of the cases were asymptomatic. However, without demographic characteristics, co-morbidities and the numbers of cases requiring hospitalisation or specialised care, the COVID-19 data does not lend itself to being a good data source. Nevertheless, there have been no reports to suggest that the hospitals have been overwhelmed with cases seeking treatment. This is in sharp contrast with what happened in Italy, Spain and America where extra facilities had to be developed urgently to meet the demand for admission and treatment space [1,3]. Additionally, case fatality was low for the reported period, approximating 1%, with 6 deaths out of 567 confirmed cases. Elsewhere, case fatality was estimated to be between 1-4%. In pandemic times, true case fatality can be confounded by other factors and thus difficult to give a true estimate. Patients who are SARS-CoV-2 PCR positive who die from other causes may end up being recorded as COVID-19 deaths.

Considering the current health sector challenges in the country, higher case fatality would have been expected. Reports indicate a fragile health infrastructure, with grossly limited capacity for intensive care and respiratory support. An upsurge in cases will overwhelm the system and threatens to further destabilise the volatile healthcare sector, as demonstrated in previous epidemics elsewhere [15]. We postulate that the majority of cases were young and healthy, as evidenced by history of travel for economic reasons. Unlike those who died in Italy, Spain and other countries, the majority of our cases had no comorbidities such as diabetes and hypertension, and were asymptomatic [16]. These co-morbidities have been the greatest risk factor for mortality elsewhere [16]. Out of the 567 confirmed cases, 142 were reported as recovered. These were spontaneous recoveries, without any specialised treatment. The definition of recovery encompassed two negative repeat RT-PCR tests from samples collected at least 24 hours apart, following guidance from the Centres for Disease Control (CDC). With the limitations in accessing repeat testing, most cases may therefore have considered active for unnecessarily long without any significant clinical symptomatology. Recently, the MOHCC adopted the WHO de-isolation criteria, which does not dictate repeat testing, and asymptomatic cases can be considered recovered after 10 days from diagnosis. Based on this new criterion, it is expected that the number of reported recoveries will rise significantly.

The study used publicly made available data and therefore did not require any extra data collection. Consequently, there were no costs incurred for the study. Unfortunately, there were several limitations to the study. There were several missing essential data elements, especially the demographics of the cases, including disaggregation by gender, age groups and co-morbidities, which would have allowed us to reconstruct a true picture of the trends. Without primary data collection, this weakness is expected. Though the MoHCC releases daily situation reports containing data on the number of positive cases for each of the 10 provinces in Zimbabwe, there are instances where some of the data has been recalled and corrected. This overall, raises questions regarding the accuracy, integrity and validity of the data. Despite a comprehensive testing strategy designed by the government, the shortage of test kits meant inadequate testing. Precisely, the testing was concentrated on points of entry and returnees, with limited community testing. This may distort the picture of the pandemic, biasing burden more towards imported cases.

We recommend that the MoHCC´s situational reports be more consistent and that there be more information on the demographic characteristics of the cases, including age and gender distribution as well as the presence of any comorbid conditions among the cases. This will inform comprehensive risk stratification and promote tailor-made interventions to protect the more vulnerable, but will also inform public health practice appropriately. Without widespread COVID-19 testing in the country, it is difficult to estimate the true burden of the disease. We recommend upscaling of testing in the 10 provinces of Zimbabwe. As evidence points towards the uncertain role of RDTs in the management of COVID-19, these must be dropped, and all efforts be made to upscale the availability of RT-PCR, which is the diagnostic gold standard. That the majority of cases have been detected among returnees calls for more vigilance and surveillance at points of entry, containment of porous borders with neighbouring countries, and initial RT-PCR of all returning residents at point of entry. This will enable timeous identification and isolation of those who test positive, before they mix with others in quarantine centres. The infection prevention and control standards in the quarantine centres must be addressed urgently by putting in place standard operating procedures, if we are to avoid these centres becoming the new hotspots for COVID-19 transmission in Zimbabwe.

## Conclusion

The trajectory of the COVID-19 outbreak in Zimbabwe so far has exhibited significant differences from what was experienced elsewhere. Caseload remains low, case fatality is around 1% and there was no evidence of community transmission. However, with the number of returnees on the rise and the quarantine system not adequately prepared to handle large numbers of returnees, Zimbabwe faces the risk of imploding into community transmission. The Zimbabwe health system, which is a key determinant of health outcomes, has been underperforming due to poor financing, low performing human resources for health, and leadership challenges. COVID-19 is therefore an added burden to a system that is struggling to address HIV infections, maternal and child health and non-communicable diseases among others. Zimbabwe needs all hands on deck, ensuring that all available human resources are supported and protected through provision of Personal Protective Equipment and a conducive working environment.

### What is known about this topic

- The COVID-19 pandemic grew exponentially in Europe, America and other parts of the world once the first cases had been reported;
- The pattern of spread in Africa has somewhat been different from what was experienced on the other continents;
- The case fatality rate of COVID-19 in other settings has been between 1-4%.

### What this study adds

- The pattern of spread in Zimbabwe between 20^th^ March and 27^th^ June 2020 was different from what was witnessed elsewhere;
- The majority of cases confirmed in Zimbabwe between 20^th^ March and 27^th^ June 2020 were imported from neighbouring countries, particularly South Africa, and Zimbabwe was not yet in community transmission;
- Efforts to contain the pandemic should therefore be appropriately aimed at containing imported cases to avoid overwhelming community transmission.
